# A Clinicopathologic Analysis of Decidual Polyps: A Potentially Problematic Diagnosis

**DOI:** 10.1155/2022/2200790

**Published:** 2022-04-11

**Authors:** Juan Zou, Ying He, Huiling Chen, Peng Wang, Xue Xiao, Shanling Liu

**Affiliations:** ^1^Department of Pathology, West China Second University Hospital, Sichuan University, Chengdu 610041, China; ^2^Key Laboratory of Birth Defects and Related Diseases of Women and Children (Sichuan University), Ministry of Education, West China Second Hospital, Sichuan University, Chengdu 610041, China; ^3^Department of Gynecology and Obstetrics, West China Second University Hospital, Sichuan University, Chengdu 610041, China; ^4^Department of Academic Affairs, West China School of Medicine, Sichuan University, Chengdu 610041, China; ^5^Department of Medical Genetics, West China Second University Hospital, Sichuan University, Chengdu 610041, China

## Abstract

**Objective:**

The decidual polyp is a special cervical polyp that is not systemically reported or well known. The aim of this study was to describe the clinicopathologic features of the decidual polyps observed at the West China Second University Hospital of Sichuan University between 2015 and 2020 and to spread awareness of them.

**Methods:**

Two hundred and fifty cases of decidual polyps, accounting for 45.45% (250/550) of all cervical polyps identified during pregnancy, were reviewed. The patients were followed up until the end of their pregnancies, which delivered <28 weeks and between 28 and 37 weeks, and full-term delivery. The *t*-test or nonparametric test was used to measure the data, and the chi-square test was used for counting data. Statistical significance was set at *p* < 0.05.

**Results:**

Most of the decidual polyps occurred during the first trimester, with a median patient age of 33 years. The polyps were both singles and multiples and located at the cervix, with a long stalk, and a median diameter of one centimeter. The gross morphological appearance varied from polypoid to lingulate, and they were fragile and bled easily. Microscopically, the decidual polyps showed diffuse glandular secretion as well as decidual changes in the stromal cells. They could be divided into two subtypes: decidua fragment and decidua with endometrial polyp formation. Seventy-three patients who went on to have further pregnancies were followed until the end of the study period. Twenty-one (21/73, 28.77%) of them had adverse pregnancy outcomes (12 cases delivered <28 weeks and 9 cases delivered between 28 and 37 weeks).

**Conclusions:**

The data showed that the decidual polyp was the second most common cervical polyp during pregnancy, and its incidence was associated with adverse pregnancy outcomes. Thus, this type of polyp should be considered in cervical polypectomy specimens from pregnant women. A more uniform and accurate pathological diagnosis, including the thrombus status and division subtype, could provide the basis for obstetricians to promote treatment improving pregnancy outcomes.

## 1. Introduction

Decidual polyps are a common condition that has not been well reported. Prior to this study, less than 100 cases have been identified [1–11]. Most of these are case reports and small sample reports. Decidual polyps mainly occur during pregnancy, especially during the early and middle stages. However, their pathogenesis is unclear. Possible reasons for their occurrence may be an endometrial overreaction stimulated by high levels of estrogen and progesterone, and an abnormality of the cervical canal, including malformation and insufficiency [1, 12]. Decidual polyp formation may be a pathologic process or a transient physiological change during pregnancy, and polyps can resolve spontaneously in some patients [1, 2]. They have been reported to increase the risk of adverse pregnant outcomes [6, 7]. Most decidual polyps are misdiagnosed as endocervical polyps by obstetricians, and currently, there is no standardized treatment for them [13]. The main treatments are polypectomy via a digital polypectomy or laser excision, or a loop electrosurgical excision procedure (LEEP). Nevertheless, conservative treatment is not frequently used [1–3, 6–11]. Errors in pathological diagnoses are often caused by limited information about the specimens, including their anatomical origin. Therefore, diagnostic terms vary widely and include decidua, endometrial polyp with decidual change, and endocervical polyp with decidual change [10]. Most studies have shown that the lesion was linked with the uterine cavity and that the pathological morphology was typical of the endometrium during pregnancy, and they also refer to interstitial decidual changes accompanied by glandular hypersecretion or secretion exhaustion [1–3, 7]. Some pathologists and obstetricians lack a unified understanding of decidual polyps, so there are discrepancies in the diagnoses. Due to the potential for decidual polyps to contribute to adverse pregnancy outcomes, it is imperative to develop a standardized pathological diagnosis.

Hence, the goal of the current study was to review all the cases of decidual polyps received in the department over a five-year period and to characterize the clinical and pathologic features of this potentially problematic condition.

## 2. Materials and Methods

### 2.1. Case Selection

A search of the pathology data dated from 2015 to 2020 at the West China Second University Hospital of Sichuan University was performed, using the keywords “cervical polyp” or “decidua” and “pregnancy.” All the available hematoxylin- and eosin-stained slides were reviewed, the cervical polyps that only contained decidua were identified, and the clinical follow-up information was confirmed. The study was approved by the ethics committee of the hospital, and informed consent was not required because the data were anonymized.

Clinical data, including age, chief complaint, gestational weeks of diagnosis at polypectomy, patients' personal medical history, complications of pregnancy, physical examination, colposcopy, ultrasonic data, and the therapeutic schedule were obtained from the electronic medical records or the referring pathologists, obstetricians, or patients.

### 2.2. Histopathology

A decidual polyp (synonymous for decidua prolapse or extruded fragment of decidua) is described as the presence of expulsed fragments of the decidua at the cervix [14]. Both the gland and stroma exhibit decidual changes. Additionally, concomitant characteristics that are exhibited include endometrial polyps, erosion, inflammation, necrosis, hemorrhage, and thrombi.

### 2.3. Statistics and Bioinformatics

The patients were followed until the end of their pregnancies, which delivered <28 weeks and between 28 and 37 weeks, and full-term delivery. SPSS 20.0 for Windows was used to analyze the data. A *t*-test or nonparametric test was used to measure the data and the chi-square test used for counting data. Statistical significance was set at *p* < 0.05 .

## 3. Results

### 3.1. Clinical Features

Between 2015 and 2020, there were 2,952 cases of cervical polyps. Of these, 550 cervical polyps occurred during pregnancy, and they included 250 cases of decidual polyps (250/550, 45.45%). The excluded 300 cases comprised 290 endocervical polyps (290/550, 72.82%) and 10 other lesions. Patient information for 33 cases of endocervical polyps was retained to compare against the decidual polyp group.

The median age of the 250 patients with decidual polyps was 33 years (ranging from 21 to 44 years). Of them, 208 cases were diagnosed in the first trimester, 40 in the second trimester, and 2 in the third trimester (overall ranging from the 4th week to the 32nd week). In addition, 42% (105/250) of the patients had a complaint of vaginal spotting. We found 8 cases of systemic comorbidities (2 cases of thrombocytopenia, 4 cases of hypothyroidism, 1 case of myasthenia gravis, 1 case of thalassemia), 20 cases of gynecological comorbidities (6 cases of uterine myoma, 4 cases of ovarian cyst, 10 cases of vaginitis), and 9 cases of pregnancy complications (7 cases of gestational diabetes, 1 case of gestational hypertension, 1 case of intrahepatic cholestasis of pregnancy). Progesterone was used in 13 pregnancies, antibiotics in 10, asthma medication in 1, and aspirin in 1. With respect to the identification of the polyps, 134 cases were diagnosed accidentally due to artificial abortion for nonmedical reasons, 43 were discovered during a curettage for embryo arrest, and the remaining 73 were followed up until pregnancy termination after a polypectomy (Table 1).

### 3.2. Ultrasonic Examination, Vaginal Cervical Examination, and Colposcopy

**Figure 1:**
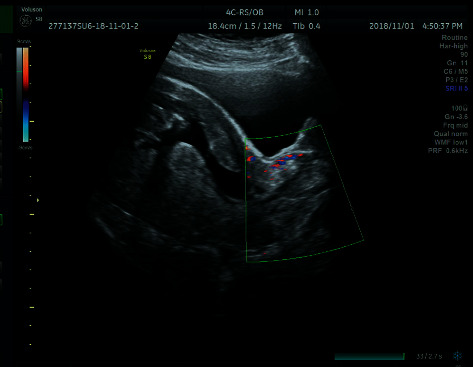
Ultrasonic images of a decidual polyp. A long weak echo was observed in the cervical canal, protruding to the external cervix with a clear boundary. The blood supply seemed to come from the posterior wall of the uterine isthmus.

**Figure 2:**
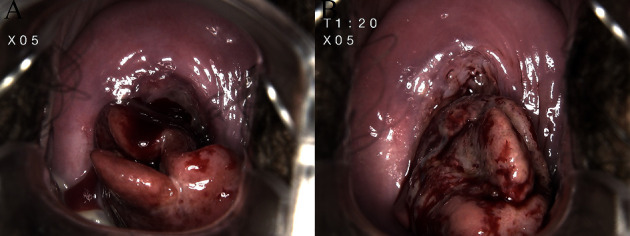
Colposcopy images of a decidual polyp: (a) an irregular lingulate polyp, 3 cm in diameter, with roots in the cervical canal, which is fragile, soft, and bleeds easily, and (b) a positive Acetowhite test.

Of the included patients, 8.8% (22/250) get a positive ultrasonography. A strip hypoechoic lesion in the cervical canal showed close relation to the uterine cavity not only through the morphological structure but also the blood supply. The positive rate of ultrasonography in term delivery group was lower than that in adverse outcome group (*p*=0.028) (Figure 1). Meanwhile, 87.20% (218/250) underwent a vaginal cervical examination, and 8.40% (21/250) underwent a colposcopy. The decidual polyps were single or multiple, ranging from one to three in number. Most of them were polypoid or lingulate with a thick stem in the cervical canal. The polyp surfaces were described as pink and fragile, and they bled easily. In some cases, the presence of the necrotic-appearing tissue was confirmed by a positive acetowhite test (Figure 2). A cervical cytology examination was performed in 85 patients, with 14.12% (12/85) having positive atypical squamous cells of an undetermined significance or low-grade squamous intraepithelial lesion results, while the remaining results were negative.

### 3.3. Treatment Schedule

All 250 patients in the study had undergone a polypectomy without anti-infection therapy. Most of them (199/250, 79.6%) underwent the polypectomy immediately after the polyp discovery, with an interval ranging from 2 to 14 weeks between the two. During this time, there was no prominent change in the polyp diameters. In total, 186 patients underwent a polypectomy in the first trimester, 62 in the second trimester, and 2 in the third trimester.

### 3.4. Pathologic Features

**Figure 3:**
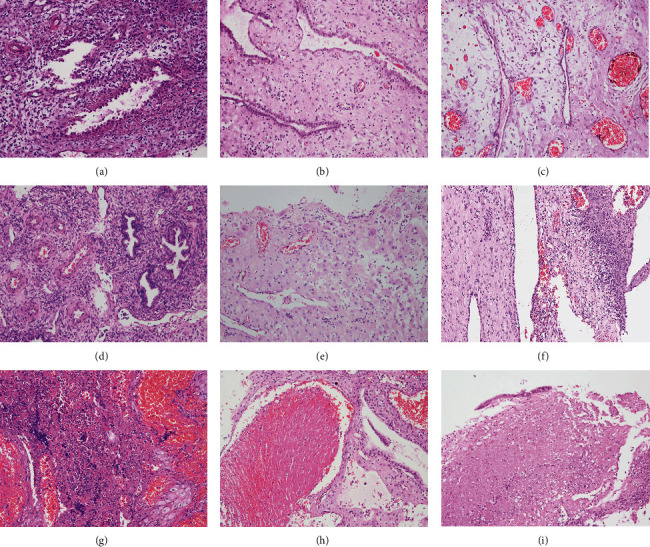
Hematoxylin and eosin staining (100×) of the decidual polyp shows the following: (a) in the first trimester, the glands show the Arias-Stella reaction with vacuolated cytoplasm and enlarged, irregular, hyperchromatic nuclei, while the stroma cells show edema and mild decidual change, and the spiral arteries proliferate; (b) in the second trimester, the gland secretions are exhausted with single-layer cuboidal epithelium, and the stroma cells show prominent decidua change with abundant pink cytoplasm and well-defined cellular borders; (c) in the third trimester, the gland secretions exhausted and extremely atrophic with single-layer flattened epithelium, and the stroma cells show edema and prominent decidual change; (d) an endometrial polyp subtype; (e) superficial erosion. (f) Acute inflammation with neutrophil granulocytes; (g) hemorrhage; (h) thrombus; and (i) necrosis.

Fifty-two patients were diagnosed with endocervical polyps at the initial diagnosis, with a misdiagnosis rate of 20.8%. Of the remaining 198 cases, the initial pathologic results were decidua (92/250, 36.80%) and endometrial polyps with decidual change (106/250, 42.40%). The surfaces of all the resection specimens were pink, exhibited erosion, and the cut surfaces were soft and fragile. The median diameter was 1 cm (ranging from 0.5 cm to 5.5 cm). Microscopically, the stroma cells developed abundant pink and edema cytoplasm with well-defined cellular borders, and the glands showed different degrees of secretory reaction from hypersecretory reaction to secretory exhaustion (Figures 3(a)–3(c)). Some of the concomitant characteristics included endometrial polyp formation (*n* = 72), erosion (*n* = 225), inflammation (*n* = 246), necrosis (*n* = 85), hemorrhage (*n* = 57), and thrombi (*n* = 42) (Figures 3(d)–3(i). The decidual polyps were divided into two groups based on the concomitant characteristics of the endometrial polyp formation: type A, decidua fragment, and type B, decidua with endometrial polyp formation. The number of deliveries in pregnancies with type A was greater than that in type B, and the polyp diameter of type B was larger than that of type A (Table 2).

### 3.5. Follow-Up and Analysis

The 73 patients whose pregnancies continued were followed until the pregnancy came to an end. Of these, 21 (21/73, 28.77%) patients experienced adverse pregnancy outcomes (12 cases delivered <28 weeks and 9 cases delivered between 28 and 37 weeks). There were no significant differences in pregnancy outcome, gestational age at polyp discovery, or premature rupture of membranes between the two subtypes (Table 3). Detailed information on the 21 patients is presented in Table 4.

**Figure 4:**
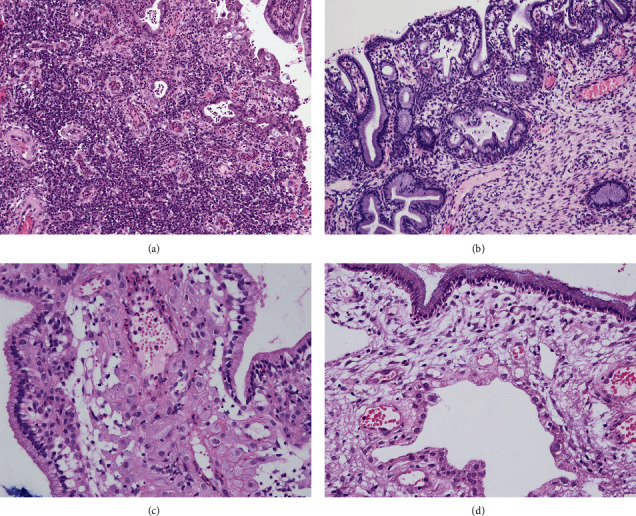
Hematoxylin and eosin staining of an endocervical polyp during pregnancy shows the following: (a) granulation tissue proliferation (100×); (b) glandular proliferation (100×); (c) stroma with focal pseudo-decidual changes (200×); and (d) a focal gestational Arias-Stella reaction, where the gland underneath has a hypersecretory reaction, compared with the superficial endocervical mucinous epithelium (200×).

A comparison was also made between the 21 patients in the decidual polyp group and 33 patients with endocervical polyps who continued their pregnancies. There were some statistically significant differences in the clinicopathological characteristics between the two groups (Table 5). The decidual polyp group had a larger polyp diameter (2.0 cm vs. 1.0 cm, *p* < 0.001), an earlier detection time (11 weeks vs. 17 weeks, *p* < 0.001), a higher proportion and a wider range of decidual-like changes (100% vs. 12.1% *p* < 0.001), and a higher rate of adverse pregnancy outcomes (28.8% vs. 6.1%, *p* < 0.05). Granulation tissue hyperplasia (81.8% vs. 6.8%, *p* < 0.001) and glandular hyperplasia (30.3% vs. 0%, *p* < 0.001) were also far more common in endocervical polyps (Figure 4).

Some pathological features were associated with a poor prognosis in the decidual polyp group with a continued pregnancy, one of them being thrombosis. The incidence of adverse outcomes was higher in patients with thrombosis than in nonthrombosis patients (83.3% vs. 16.7%, *p* < 0.05), and it may well be the main pathological feature that affects prognosis (Table 6).

## 4. Discussion

This is the largest retrospective study on decidual polyps, and, according to the data, the decidual polyp is the second most common cervical polyp during pregnancy and is related to adverse pregnancy outcomes. There was a statistically significant difference in decidual polyps complicated with gynecological diseases in different pregnancy outcomes (*p*=0.013), vaginal inflammation is the main one among these gynecological diseases, and there may be a correlation between inflammation and the development of decidual polyps. It accounts for 45.45% (250/550) of all cervical polyps during pregnancy and is consistent with the proportion of 41.41% (41/99) reported in other research [6]. However, the decidual polyp has a lower rate of diagnosis because the initial pathologic impressions are typically erroneous or inaccurate. The results therefore suggest that decidual polyps are a common but easily overlooked condition during pregnancy, requiring more attention and further research by obstetricians and pathologists.

Though often mistaken for endocervical polyps, the prognosis of endocervical polyps and decidual polyps is not the same. Adverse pregnancy outcomes were higher in the decidual polyp group than the endocervical polyp group (28.7% vs. 6.1%). It has already been reported that there is a difference between decidual polyps and endocervical polyps in delivered<28 weeks (12.2% vs. 0%) and 28–37 weeks delivery (34.2% vs. 4.8%) [6], and several studies have concluded that decidual polyps are associated with adverse pregnancy outcomes [3, 6, 7, 11]. The higher risk of adverse pregnancy outcomes after a polypectomy was further confirmed by Tokunaka (19/41, 46.4%) and Fukuta (19/52, 36.54%) [6, 7], and Zhang reported an adverse pregnancy outcome in six (6/11, 54.5%) cases with anti-inflammatory conservative management [11]. Due to the increased risk of adverse pregnancy outcomes, the decidual polyp should be treated with care.

In this study, decidual polyps were divided into two subtypes, namely decidua fragment, type A, and decidua with endometrial polyp formation, type B, and it was found that the adverse pregnancy outcome rate of type A (18/54, 33.33%) was higher than that of type B (3/18, 16.67%) (*p*=0.408) although the difference was not statistically significant. It is interesting that there was no case of type A in the third trimester, and it seems that this subtype may have a different pathogenesis. In addition, the number of births with type A was greater than the number with type B, and the polyp diameter of type B was larger than that of type A. It seems clear that more detailed classification and research on endometrial polyp formation could enhance the understanding of decidual polyps, and further research on this condition to improve pregnancy outcomes is highly recommended.

In the cases cited in this study, the initial pathologic impressions were erroneous or inaccurate, which caused a low diagnosis rate of decidual polyps. A total of 52 (52/250 20.80%) cases were misdiagnosed as endocervical polyps with decidual change. The remaining cases were inaccurately diagnosed as decidua (92/250, 36.80%) and endometrial polyps with decidual change (106/250, 42.40%). However, these results contradict those of some reports. One paper indicated that the diagnostic rate was higher [6] and maintained that since the endocervical polyp with decidual change was difficult to distinguish, an endocervical polyp with decidual change had been categorized as a decidual polyp. The lack of concern and recognition with regard to decidual polyps may be attributed to the diagnostic problem. It is, thus, necessary to sort out the pathological manifestations of decidual polyps and facilitate a unified diagnosis to improve the diagnosis rate.

Currently, the decidual polyp is rarely reported in pathology books and articles, and, when it is, it is just described as the presence of expulsed fragments of the decidua at the cervix [14], and there is no additional information. From the cases reviewed in this study, it can be inferred that the decidual polyp originates from the endometrium in the uterine body and breaks through the cervical canal to the cervix. A diagnosis of decidual polyps should be considered in cervical polypoid specimens when they (1) are taken from pregnant women; (2) have a flaky and pink surface, characterized by softness, fragility, and easy bleeding; (3) exhibit extensive decidual changes in the stromal cells; (4) demonstrate epithelium showing high progesterone reaction with high secretion or atrophic reaction; and (5) show no placental villus and trophocytes.

What follows are suggestions for the pathological diagnosis of cervical polyps during pregnancy: (1) the tissue origin should be clearly identified as endometrial or endocervical. Based on the findings of this study, the endometrial tissue is fragile and bleeds easily, whereas the cervical tissue is smooth and firm. Under the microscope, the endometrial tissue shows diffuse decidual reaction of stroma and epithelium, whereas the cervical tissue usually shows focal decidual reaction with more or less mucous epithelium and myofibrillar mesenchymal left. The granulation tissue and glandular proliferation are additional evidence for endocervical polyps. (2) For the endometrial origin, further examination of the placental villus and trophoblast components should be performed to exclude spontaneous abortion, and if none of the above mentioned components are present, a diagnosis of decidual polyps can be made. (3) Decidual polyps should be divided into two subtypes: type A, decidua fragment, and type B, decidua with endometrial polyp formation. (4) A description of the polyp should include the concomitant characteristics of endometrial polyps, namely, erosion, inflammation, necrosis, hemorrhage, and thrombi. In particular, attention should be paid to thrombi as the incidence of adverse pregnancy outcomes is higher in patients with thrombosis.

To the best of our knowledge, this is the largest study of decidual polyps, and this is the first study concerned with the pathological diagnosis of decidual polyps. It is not only the identification of decidual polyps that is important but also making an accurate pathological diagnosis. Several limitations must be considered with respect to this study. The samples were drawn from a single center, all the patients underwent a polypectomy, and there was no conservative treatment control group; therefore, the natural pathogenesis of the decidual polyp is not yet clear.

## 5. Conclusion

In summary, the decidual polyp is a common condition associated with adverse pregnancy outcomes. Despite this, it is often ignored, and universal recognition is limited. The findings of this study would suggest that a more uniform and accurate pathological diagnosis is necessary, and there should be more focus on the possibility of thrombosis and on the particular subtype due to the potential links with adverse pregnancy. The limitations of this study mean that further investigation into the pathogenesis of decidual polyp is required, along with an appropriate therapeutic schedule, to reduce the adverse pregnancy outcomes associated with this condition.

## Figures and Tables

**Table 1:** Summary of the clinic-pathological features of the decidual polyp.

		Term birth (*n* = 52)	Delivered 28–37 weeks (*n* = 9)	Delivered <28 weeks (*n* = 12)	*p*	Artificial abortion (*n* = 134)	Embryo arrest (*n* = 43)	Total (*n* = 250)
Clinical information	Age (y)	31 (27.5, 33.5)	32 (30, 35)	31.5 (29.5, 33.5)	0.116	33 (30, 37)	34 (30, 37)	33 (29.75, 36)
	G	2 (1, 2)	2 (1, 2)	2 (1, 2.5)	0.634	2 (2, 3)	2 (1,3)	2 (1, 3)
	P	0 (0,1)	1 (0,1)	1 (0,1)	0.825	1 (1, 1)	0.5 (0, 1)	1 (0, 1)
	Systemic comorbidities	6 (2.4%)	0 (0%)	2 (0.8%)	0.466	0 (0%)	0 (0%)	8 (3.2%)
	Gynecological comorbidities	6 (2.4%)	0 (0%)	5 (2.0%)	0.013	8 (3.2%)	1 (0.4%)	20 (8%)
	Medication history	7 (2.8%)	1 (0.4%)	2 (0.8%)	0.931	9 (3.6%)	6 (2.4%)	25 (10%)
	Pregnancy complications	6 (2.4%)	2 (0.8%)	0 (0%)	0.264	1 (0.4%)	0 (0%)	9 (3.6%)
	Colporrhagia	41 (16.4%)	7 (2.8%)	8 (3.2%)	0.665	34 (13.6%)	15 (6%)	105 (42%)
	Ultrasonic	1 (0.4%)	2 (0.8%)	2 (0.8%)	0.028	13 (5.2%)	4 (1.6%)	22 (8.8%)
	Leucorrhea	15 (6%)	1 (0.4%)	3 (1.2%)	0.532	42 (16.8%)	9 (3.6%)	69 (27.6%)
	Blood routine	17 (6.8%)	2 (0.8%)	4 (1.6%)	0.814	62 (24.8%)	22 (8.8%)	107 (42.8%)
	Colposcopy	13 (5.2%)	3 (1.2%)	3 (1.2%)	0.867	0 (0%)	0 (0%)	19 (7.6%)
Diagnosis information	Cervical cytology	4 (1.6%)	2 (0.8%)	3 (1.2%)	0.163	3 (1.2%)	0 (0%)	12 (4.8%)
	Discovery by accident	4 (1.6%)	0 (0%)	1 (0.4%)	0.683	82 (32.8%)	23 (9.2%)	110 (44%)
	Discovery week (w)	11.5 (9, 15.5)	16 (9, 16)	8 (5.5, 11)	0.033	6 (6, 7)	8 (7.75, 9)	7 (6, 9)
	The first trimester	21 (8.4%)	4 (1.6%)	8 (3.2%)		133 (53.2%)	42 (16.8%)	208 (83.2%)
	The second trimester	30 (12%)	4 (1.6%)	4 (1.6%)		1 (0.4%)	1 (0.4%)	40 (16%)
	The third trimester	1 (0.4%)	1 (0.4%)	0 (0%)		0 (0%)	0 (0%)	2 (0.8%)
Histopathological features	No. of polyp.	1 (1,1)	1 (1,1)	1 (1,1)	0.817	1 (1,1)	1 (1,1)	1 (1,1)
	Diameter of polyp	2 (1.5, 3)	1.5 (1, 2.5)	2.5 (1.25, 4)	0.574	1 (0.5, 1.5)	0.5 (0.5, 1)	1 (0.5, 2)
	Endometrial polyp	15 (6%)	1 (0.4%)	3 (1.2%)	0.532	42 (16.8%)	11 (4.4%)	72 (28.8%)
	Endometrial fragment	37 (14.8%)	8 (3.2%)	9 (3.6%)	0.532	92 (36.8%)	32 (12.8%)	178 (71.2%)
	Inflammation	52 (20.8%)	9 (3.6%)	12 (4.8%)	NA	131 (52.4%)	42 (16.8%)	246 (98.4%)
	Hemorrhage	15 (6%)	3 (1.2%)	3 (1.2%)	0.916	31 (12.4%)	5 (2.0%)	57 (22.8%)
	Erosion	49 (19.6%)	9 (3.6%)	11 (4.4%)	0.698	119 (47.6%)	37 (14.8%)	225 (90%)
	Necrosis	20 (8%)	4 (1.6%)	3 (1.2%)	0.606	46 (18.4%)	12 (4.8%)	85 (34%)
	Thrombus	2 (0.8%)	2 (0.8%)	3 (1.2%)	0.031	32 (12.8%)	3 (1.2%)	42 (16.8%)
	Original diagnosis							
	Decidua	23 (9.2%)	1 (0.4%)	5 (2.0%)	0.171	43 (17.2%)	20 (8%)	92 (36.8%)
	Endometrial polyp	23 (9.2%)	7 (2.8%)	4 (1.6%)	0.106	59 (23.6%)	13 (5.2%)	106 (42.4%)
	Endocervical polyp	6 (2.4%)	1 (0.4%)	3 (1.2%)	0.460	32 (12.8%)	10 (4%)	52 (20.8%)

G, gravid; P, pregnancy.

**Table 2:** Comparison of the clinic-pathological features between the two subtypes of the decidual polyp.

		Decidua fragment (*n* = 178)	Decidua with endometrial polyp formation (*n* = 72)	*p*
Age (y)		33 (30, 36)	32 (29, 36)	0.231
G		2 (2, 3)	2 (1, 3)	0.12
P		1 (0, 1)	1 (0, 1)	0.018
No. of polyp.		1 (1, 1)	1 (1, 1)	0.805
Diameter of polyp		1 (0.5, 1.5)	2 (1, 3)	<0.001
Discovery week (w)		8 (6, 9.25)	7 (6, 9)	0.081
Inflammation	Negative	3 (1.7%)	1 (1.4%)	
	Positive	175 (98.3%)	71 (98.6%)	
				0.866
Hemorrhage	Negative	144 (80.9%)	49 (68.1%)	
	Positive	34 (19.1%)	23 (31.9%)	
				0.028
Thrombus	Negative	152 (85.4%)	56 (77.8%)	
	Positive	26 (14.6%)	16 (22.2%)	
				0.15
Erosion	Negative	21 (11.8%)	4 (5.6%)	
	Positive	157 (88.2%)	68 (94.4%)	
				0.136
Necrosis	Negative	116 (65.2%)	49 (68.1%)	
	Positive	62 (34.8%)	23 (31.9%)	
				0.663

**Table 3:** Clinical outcomes of 73 patients with the decidual polyp of continuing pregnancy.

	Pregnancy outcomes				Discovery week				Premature rupture of membrane		
	Term birth	Delivery week (28–37 w)	Delivery week (<28 w)	*p*	The first trimester	The second trimester	The third trimester	*p*	Negative	Positive	*p*
Decidual fragment (*n* = 55)	37 (67.3%)	8 (14.5%)	10 (18.2%)		25 (45.5%)	30 (54.5%)	0 (0%)		48 (87.3%)	7 (12.7%)	
Decidua with endometrial polyp formation (*n* = 18)	15 (83.3%)	1 (5.6%)	2 (11.1%)		7 (38.9%)	9 (50.0%)	2 (11.1%)		16 (88.9%)	2 (11.1%)	
				0.408				0.093			1

**Table 4:** Case presentation of decidual polyps with adverse outcomes.

No.	Age (y)	G	*P*	Discovery week (w)	Dimeter (cm)	PROM	Delivery week (w)	Endometrial polyp	Inflammation	Hemorrhage	Thrombus	Erosion	Necrosis
1	34	4	1	16	2	NO	21	NO	YES	NO	NO	YES	NO
2	35	2	1	16	1.5	YES	28 + 2	NO	YES	NO	NO	YES	YES
3	37	3	1	9	1.5	NO	34 + 5	NO	YES	NO	NO	YES	NO
4	32	1	0	9	2.5	YES	33 + 2	NO	YES	YES	YES	YES	YES
5	30	3	1	16	2	NO	32	NO	YES	NO	NO	YES	NO
6	32	1	0	32	4	YES	32	YES	YES	NO	YES	YES	YES
7	34	5	1	8	0.5	YES	21 + 4	NO	YES	NO	NO	YES	NO
8	32	1	0	8	5	NO	24 + 1	YES	YES	NO	NO	YES	NO
9	29	1	0	13	5	NO	16	NO	YES	NO	NO	YES	NO
10	30	2	0	6	3	YES	15	YES	YES	NO	NO	YES	YES
11	30	1	0	5	1	YES	26 + 6	NO	YES	NO	NO	YES	YES
12	30	1	0	17	1.5	YES	34 + 1	NO	YES	YES	NO	YES	NO
13	34	2	1	5	3.7	YES	34 + 4	NO	YES	NO	NO	YES	NO
14	42	2	1	8	2	NO	36 + 1	NO	YES	NO	NO	YES	NO
15	31	2	1	5	3	YES	22 + 3	NO	YES	YES	YES	YES	NO
16	33	3	1	7	1	NO	8	NO	YES	NO	YES	YES	NO
17	33	2	1	14	2	YES	20 + 5	NO	YES	NO	NO	YES	NO
18	28	1	0	7	0.4	NO	24	NO	YES	NO	NO	YES	NO
19	29	2	1	9	5	YES	20	NO	YES	YES	NO	YES	YES
20	30	1	0	16	0.5	NO	35	NO	YES	YES	NO	YES	YES
21	38	2	1	9	3	NO	24	NO	YES	YES	YES	YES	YES

G, gravid; P, pregnancy; PROM, preterm rapture of membrane.

**Table 5:** Comparison of endocervical polyps and decidual polyps in continuing pregnancy.

		Endocervical polyp (*n* = 33)	Decidual polyp (*n* = 73)	*p*
Age (y)		30 (28, 33)	31 (29, 34)	0.447
G		1 (1, 2)	2 (1, 2)	0.117
P		0 (0, 1)	0 (0, 1)	0.032
Diameter of polyp		1 (00.75, 1.650)	2 (1.5, 3.0)	<0.001
Discovery week (w)		17 (12.5, 22.5)	11 (8, 16)	<0.001
Pregnancy outcomes	Term birth	31 (93.9%)	52 (71.2%)	
	Adverse outcomes	2 (6.1%)	21 (28.8%)	0.018
Inflammation	Negative	0	0	
	Positive	33 (100%)	73 (100%)	1
			
Hemorrhage	Negative	29 (87.9%)	54 (74.0%)	
	Positive	4 (12.1%)	19 (26.0%)	
				0.108
Thrombus	Negative	30 (90.9%)	67 (91.8%)	
	Positive	3 (9.1%)	6 (8.2%)	
				0.881
Erosion	Negative	5 (15.2%)	2 (2.7%)	
	Positive	28 (84.8%)	71 (97.3%)	
				0.05
Necrosis	Negative	26 (78.8%)	47 (64.4%)	
	Positive	7 (21.2%)	26 (35.6%)	
				0.138
Decidual change	Negative	29 (87.9%)	0 (0%)	
	Positive	4 (12.1%)	100 (100%)	<0.001
			
Granulation hyperplasia	Negative	6 (18.2%)	68 (93.2%)	
	Positive	27 (81.8%)	5 (6.8%)	
				<0.001
Glandular hyperplasia	Negative	23 (69.7%)	100 (100%)	
	Positive	10 (30.3%)	0 (0%)	
				<0.001

**Table 6:** Relationship between different pathological morphologies and adverse outcomes.

		Adverse outcomes (*n* = 21)	Term birth (*n* = 52)	*p*
Inflammation	Negative	0 (0%)	0 (0%)	
	Positive	21 (100%)	52 (100%)	
				1
Hemorrhage	Negative	15 (27.8%)	39 (72.2%)	
	Positive	6 (31.6%)	13 (68.4%)	
				0.753
Thrombus	Negative	16 (23.9%)	51 (76.1%)	
	Positive	5 (83.3%)	1 (16.7%)	
				0.009
Erosion	Negative	0 (0%)	2 (100%)	
	Positive	21 (29.6%)	50 (70.4%)	
				1
Necrosis	Negative	13 (27.7%)	34 (72.3%)	
	Positive	8 (30.8%)	18 (69.2%)	
				0.779

## Data Availability

The authors declare that materials described in the manuscript, including all relevant raw data, will be freely available to researchers, without breaching participant confidentiality.
